# Clinical associations with stage B heart failure in adults with type 2 diabetes

**DOI:** 10.1177/20420188211030144

**Published:** 2021-07-17

**Authors:** Gaurav S. Gulsin, Emer Brady, Anna-Marie Marsh, Gareth Squire, Zin Z. Htike, Emma G. Wilmot, John D. Biglands, Peter Kellman, Hui Xue, David R. Webb, Kamlesh Khunti, Tom Yates, Melanie J. Davies, Gerry P. McCann

**Affiliations:** Department of Cardiovascular Sciences, University of Leicester, Glenfield Hospital, Groby Road, Leicester, LE39QP, UK; Department of Cardiovascular Sciences, University of Leicester and the National Institute for Health Research (NIHR) Leicester Biomedical Research Centre, Leicester, UK; Department of Cardiovascular Sciences, University of Leicester and the National Institute for Health Research (NIHR) Leicester Biomedical Research Centre, Leicester, UK; Department of Cardiovascular Sciences, University of Leicester and the National Institute for Health Research (NIHR) Leicester Biomedical Research Centre, Leicester, UK; Diabetes Research Centre, University of Leicester and the NIHR Leicester Biomedical Research Centre, UK; Diabetes Department, Royal Derby Hospital, University Hospitals of Derby and Burton NHS Foundation Trust, Derby, UK; NIHR Leeds Biomedical Research Centre, Leeds, UK; National Heart, Lung and Blood Institute, Bethesda, MD, USA; National Heart, Lung and Blood Institute, Bethesda, MD, USA; Diabetes Research Centre, University of Leicester and the NIHR Leicester Biomedical Research Centre, UK; Diabetes Research Centre, University of Leicester and the NIHR Leicester Biomedical Research Centre, UK; Diabetes Research Centre, University of Leicester and the NIHR Leicester Biomedical Research Centre, UK; Diabetes Research Centre, University of Leicester and the NIHR Leicester Biomedical Research Centre, UK; Department of Cardiovascular Sciences, University of Leicester and the National Institute for Health Research (NIHR) Leicester Biomedical Research Centre, Leicester, UK

**Keywords:** stype 2 diabetes, heart failure, diabetic cardiomyopathy, risk factors, cardiovascular magnetic resonance imaging

## Abstract

**Background:**

There is a high prevalence of asymptomatic (American Heart Association Stage B) heart failure (SBHF) in people with type 2 diabetes (T2D). We aimed to identify associations between clinical characteristics and markers of SBHF in adults with T2D, which may allow therapeutic interventions prior to symptom onset.

**Methods:**

Adults with T2D from a multi-ethnic population with no prevalent cardiovascular disease [*n* = 247, age 52 ± 12 years, glycated haemoglobin A1c (HbA1c) 7.4 ± 1.1% (57 ± 12 mmol/mol), duration of diabetes 61 (32, 120) months] underwent echocardiography and adenosine stress perfusion cardiovascular magnetic resonance imaging. Multivariable linear regression analyses were performed to identify independent associations between clinical characteristics and markers of SBHF.

**Results:**

In a series of multivariable linear regression models containing age, sex, ethnicity, smoking history, number of glucose-lowering agents, systolic blood pressure (BP) duration of diabetes, body mass index (BMI), HbA1c, serum creatinine, and low-density lipoprotein (LDL)-cholesterol, independent associations with: left ventricular mass:volume were age (β = 0.024), number of glucose-lowering agents (β = 0.022) and systolic BP (β = 0.027); global longitudinal strain were never smoking (β = −1.196), systolic BP (β = 0.328), and BMI (β = −0.348); myocardial perfusion reserve were age (β = −0.364) and male sex (β = 0.458); and aortic distensibility were age (β = −0.629) and systolic BP (β = −0.348). HbA1c was not independently associated with any marker of SBHF.

**Conclusions:**

In asymptomatic adults with T2D, age, systolic BP, BMI, and smoking history, but not glycaemic control, are the major determinants of SBHF. Given BP and BMI are modifiable, these may be important targets to reduce the development of symptomatic heart failure.

## Background

In asymptomatic individuals with type 2 diabetes (T2D) there is a high prevalence of left ventricular (LV) systolic and diastolic dysfunction and cardiac remodelling.^1,2^ The American Heart Association has classified such individuals as having stage B heart failure (SBHF), which describes structural or functional heart disease in the absence of current or prior heart failure symptoms.^3^ Isolated abnormalities of LV diastolic dysfunction and reduced global longitudinal strain (GLS) are associated with incident heart failure in T2D. Their identification may permit earlier recognition and treatment of patients most at risk of heart failure. This is especially significant given the vast majority of people with T2D develop heart failure with preserved ejection (HFpEF), a condition that has no proven effective treatments.^4^ We have recently demonstrated that subclinical concentric LV remodelling, arterial stiffening, abnormalities in diastolic and systolic function, and reduced myocardial perfusion are present in T2D,^5,6^ and these features are increasingly recognised as pathognomic features of SBHF and precursors to the onset of clinical heart failure.^4,5^ The clinical factors contributing to early cardiovascular dysfunction in people with T2D, however, are poorly understood. Previous imaging studies have been hindered by variations in methods of assessing cardiac structure and function, as well as small sample sizes (seldom greater than 100 subjects) and incomplete datasets with significant risk of overfitting the regression models.^4^

Cardiovascular magnetic resonance (CMR) is the gold standard imaging modality for assessment of cardiac volumes and mass, and with the addition of stress perfusion imaging has the ability to provide accurate quantification of myocardial blood flow (MBF). By combining CMR with echocardiography, comprehensive cardiovascular phenotyping of asymptomatic adults with T2D is possible.

**Figure 1.**
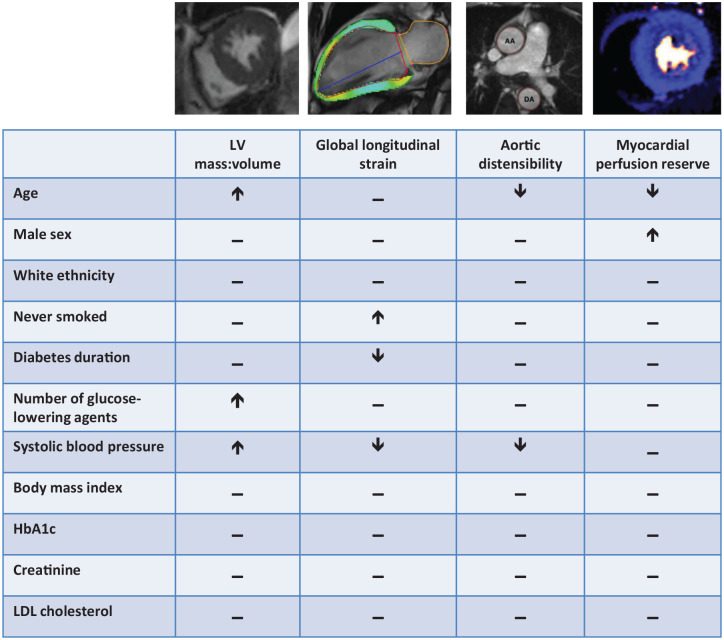
There is a high prevalence of asymptomatic SBHF in people with T2D, but the clinical contributors to early heart failure in these individuals are not clearly understood. We undertook a series of multivariable linear regression analyses in 247 adults with T2D from a multi-ethnic population with no prevalent cardiovascular disease, who underwent echocardiography and adenosine stress perfusion CMR imaging. This was to identify independent associations between clinical characteristics and markers of SBHF (concentric left ventricular remodelling, reduced global longitudinal strain, increased aortic stiffness, and impaired myocardial perfusion reserve). Age, systolic blood pressure, BMI and smoking history, but not glycaemic control, were the major determinants of SBHF. BMI, body mass index; CMR, cardiovascular magnetic resonance; SBHF, stage B heart failure; T2D, type 2 diabetes.

The aim of this study was to identify independent associations between clinical characteristics and key cardiovascular perturbations typical of SBHF in a multi-ethnic cohort of asymptomatic adults with T2D (Figure 1).

## Research design and methods

### Participants

This was a pooled analysis of individual baseline patient data from participants recruited to one of four studies evaluating the impact of T2D on cardiovascular structure and function (Table 1).^7–10^ Adults with T2D were enrolled prospectively into these studies from primary and specialist care services in Leicestershire, UK, with support from the National Institute for Health Research (NIHR) East Midlands Clinical Research Network. Participants included in the current analyses were aged 18–75 years, with no prior history, clinical signs or symptoms of cardiovascular disease and no contraindications to CMR imaging. Exclusion criteria were: type 1 diabetes, stage 4 or 5 chronic kidney disease (estimated glomerular filtration rate <30 ml/min/1.73 m^2^), known macrovascular disease (including myocardial infarction, transient ischaemic attack, stroke, peripheral artery disease), presence of arrhythmia, history of heart failure, moderate or severe valvular heart disease, and cardiovascular symptoms (such as angina or limiting dyspnoea during normal physical activity). Ethical approval for each study was granted by the National Research Ethics Service, conducted according to the Declaration of Helsinki, and all participants provided written informed consent prior to any testing.

**Table 1.** Overview of studies from which participant baseline data was pooled. For the present analyses, subjects with a history, signs or symptoms of cardiovascular disease were excluded.

Study title and acronym	Funding	Trial registry/REC reference	Key inclusion/exclusion criteria	Synopsis
The Emerging Epidemic of Type 2 Diabetes in Young Adults – the EXPEDITION study^9^	Medical Research Council Interdisciplinary Bridging Award	ISRCTN: 60207691 REC: North Nottinghamshire, 09/H0407/9	Inc: stable T2D, age 18–39 y. Exc: angina or limiting dyspnoea (>NYHA class II), history of CVD, arrhythmia, moderate or worse heart valve disease, eGFR < 30 mL/min/1.7 m^2^.	Case-control study to assess the cardiovascular, anthropometric and biochemical determinants of diastolic dysfunction in young adults with T2D using multiparametric CMR.
Effects of Liraglutide in Young Adults with Type 2 Diabetes – the LYDIA study^8^	Jointly funded by Leicester-Loughborough Diet, Lifestyle and Physical Activity Biomedical Research Unit and by Novo Nordisk.	[ClinicalTrials.gov identifier: NCT02043054] REC: West Midlands, 13/WM/0311	Inc: stable T2D, age 18–50 y, BMI ⩾30 kg/m^2^ (⩾27 kg/m^2^ if south Asian or BME), A1c 6.5–10%. Exc: eGFR < 30 mL/min/1.73 m^2^, active CVD including MI within past 6 months and/or heart failure (NYHA class III and IV), on GLP-1RA or DPPIVi.	Open-label, randomised, active-comparator trial investigating the cardiometabolic effects of Liraglutide (a GLP-1RA) compared with that of its clinically relevant comparator Sitagliptin (a DPPIVi) in young adults with T2D.
Diabetes Interventional Assessment of Slimming or Training to Lessen Inconspicuous Cardiovascular Dysfunction – the DIASTOLIC study^7^	National Institute for Health Research Career Development Fellowship (G. P. McCann)	[ClinicalTrials.gov identifier: NCT02590822] REC: West Midlands, 15/WM/0222	Inc: stable T2D, age 18–65 y, BMI ⩾30 kg/m^2^ (⩾27 kg/m^2^ if south Asian or BME), A1c 6.5–10%. Exc: T2D duration >12 y, angina or limiting dyspnoea (>NYHA class II), history of CVD, arrhythmia, moderate or worse heart valve disease, eGFR < 30 mL/min/1.73 m^2^.	PROBE trial with nested case-control study: 1) to determine the cause of diastolic dysfunction, assessed by CMR, in young adults with T2D and 2) determine if diastolic dysfunction can be reversed by either a low energy meal replacement diet or an exercise programme.
Prevalence and Determinants of Subclinical Cardiovascular Dysfunction in Adults with Type 2 Diabetes – the PREDICT study^10^	British Heart Foundation Clinical Research Training Fellowship (G. S. Gulsin)	[ClinicalTrials.gov identifier: NCT03132129] REC: West Midlands, 17/WM/0192	Inc: age 18–75 y. Exc: angina or limiting dyspnoea (>NYHA class II), history of CVD, arrhythmia, moderate or worse heart valve disease, eGFR < 30 mL/min/1.73 m^2^.	Cross-sectional study to identify the prevalence of subclinical cardiovascular dysfunction and identify the key aetiological factors in adults with T2D.

BME, black or other minority ethnicity; CMR, cardiovascular magnetic resonance imaging; DPPIVi, dipeptidyl peptidase-IV inhibitor; exc:, exclusion critera; GLP-1RA, glucagon-like peptide-1 receptor agonist; Inc:., inclusion critera; ISRCTN, International Standard Randomised Controlled Trials Number; PROBE, prospective, randomised, open-label blinded-endpoint; REC, research ethics committee; T2D, type 2 diabetes.

### Assessments

Demographics, medical history, and anthropometric measures were collected at the assessment visits. Glucose-lowering therapies taken by participants were categorised as: 0 = none, 1 = one oral agent; 2 = two oral agents; 3 = three or more oral agents, and 4 = insulin-treated. Smoking history was categorised as: never, ex-smoker, or current smoker. A fasting blood sample was collected for biochemical profile for diabetes control, lipids, liver, and kidney function.

#### Transthoracic echocardiography

Transthoracic echocardiography was performed in a subset of participants (*n* = 175), by two accredited operators using an iE33 system with S5-1 transducer (Philips Medical Systems, Best, The Netherlands). Images were acquired and reported as per American Society of Echocardiography guidelines.^11^ Early diastolic transmitral flow velocities (E) and early diastolic mitral annular velocities (e′) to estimate LV filling pressures were assessed by Doppler echocardiography per current recommendations.^12^

#### CMR imaging

CMR scanning was performed using a standardised protocol on Siemens scanners (Erlangen, Germany) at either 1.5T (Siemens Aera) or 3T (Siemens Skyra). In brief, after localisers, steady-state free precession cine images were acquired in four-, three- and two-chamber views. Perfusion images were then acquired after vasodilatory stress with adenosine (140 μg/kg/min, infused intravenously for 3 min). At peak stress, a gadolinium-based contrast agent was injected followed by a 20 ml bolus of normal saline, at a rate of 5 ml/s, and perfusion images were acquired at three short-axis slices (basal, mid and apical). Rest imaging was performed approximately 10 min after stress. In between rest and stress imaging, a stack of short-axis slices was obtained using cine images to obtain coverage of the entire LV. For measurement of aortic distensibility, steady-state free precession aortic cine images were acquired in a plane perpendicular to the thoracic aorta at the level of the pulmonary artery bifurcation. Simultaneous brachial blood pressure (BP) was measured using an automatic oscillometric device. Late gadolinium enhancement (LGE) images were acquired approximately 10 min after the rest perfusion contrast dose for assessment of focal myocardial fibrosis.

#### Image analysis

Each CMR image set was assigned a unique study identifier using an independent online computerized random number generator. CMR images were analysed offline blinded to all patient details. Cardiac chamber volumes, function and strain were assessed by a single experienced observer (G.S.G) using cmr42 version 11 (Circle Cardiovascular Imaging, Calgary, Alberta, Canada). Myocardial strain measurement was performed using cmr42 Tissue Tracking from balanced steady-state free-precession long axis cine images (to calculate GLS). Perfusion images were assessed qualitatively for focal and subendocardial perfusion defects, and individuals with reversible perfusion defects indicative of ischaemia due to epicardial coronary artery disease were excluded from further analyses. Quantitative myocardial perfusion analysis was performed using a saturation recovery gradient echo pulse sequence (at 1.5T),^9^ with signal intensity versus time curves converted to concentration curves using a linear signal response to contrast agent with Fermi-constrained deconvolution or using a dual sequence gradient echo method with inline automated reconstruction and post-processing for MBF quantification (at 3T) at base, mid and apical slice positions.^13,14^ Stress and rest MBF at basal, mid and apical slices were averaged to derive global stress and rest MBF, and determine myocardial perfusion reserve (MPR, calculated as global stress MBF/global rest MBF). Aortic distensibility was analysed by two experienced operators (KP and GS) using Java Image Manipulation version 6 (Xinapse Software, Essex, UK) as previously described.^15^ LGE images were assessed for focal fibrosis (GSG and GPM), categorised as present or absent, and individuals with a subendocardial pattern of late enhancement indicative of previous myocardial infarction were excluded from further analyses.

### Statistical analysis

Normality was assessed using histograms. Continuous data are expressed as mean [± standard deviation (SD)], if normally distributed or median (interquartile range) if not.

Correlations between clinical characteristics (age, diabetes duration, glucose-lowering therapies, body weight, BMI, BP, heart rate, biochemistry), and CMR and echocardiographic measures of cardiovascular structure and function (LV mass:volume, systolic and diastolic function, MPR, and aortic distensibility) were assessed using Spearman’s rank correlation coefficient.

Generalised linear modelling was performed to identify independent associations between clinical characteristics with measures of cardiovascular structure and function; specifically LV mass:volume, GLS, MPR, aortic distensibility, E/A ratio and average E/e′. A separate model, with each CMR and echocardiographic measure as the dependent variable, tested individually against a combination of key clinical characteristics presumed to be associated with diabetic cardiomyopathy,^16^ was performed. The clinical variables included in each model were age, sex, ethnicity, smoking history, systolic BP, duration of diabetes, glucose-lowering therapies, BMI, HbA1c, serum creatinine and LDL cholesterol. Continuous predictor variables were standardised prior to inclusion in the regression models to permit more direct comparisons of the magnitude of their effects on the dependent variable. Categorical variables were set to have the largest group defined as the reference. Regression coefficients (β) are presented as point estimate and 95% confidence intervals (CIs). To minimise the risk of overfitting our regression models, multiple imputation was used to impute missing CMR and echocardiography data, which has been shown to be a valid general method for handling missing data.^17^

Statistical analysis was performed using SPSS version 25.0 (Statistical Package for Social Sciences, Chicago, IL, USA). A *p* value < 0.05 was considered statistically significant.

## Results

**Figure 2.**
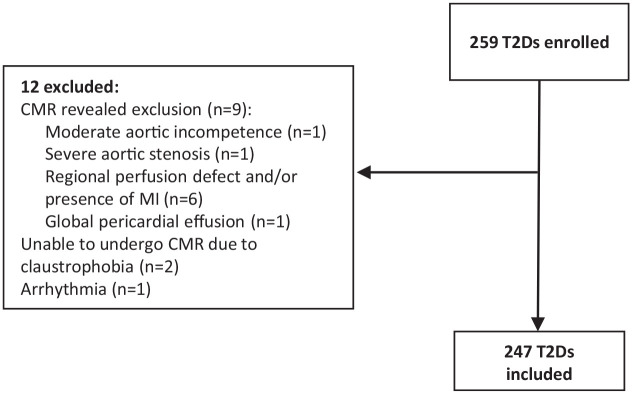
Study profile. CMR, cardiovascular magnetic resonance; MI, myocardial infarction; T2D, type 2 diabetes.

The study profile is displayed in Figure 2. At baseline 259 subjects with T2D were recruited; 12 subjects with T2D were excluded after consent and reasons for ineligibility are shown in Figure 2. A total of 247 subjects with T2D were therefore included in the analyses.

### Participant characteristics

The baseline demographic characteristics of subjects with T2D are shown in Table 2. Mean age of participants was 51.8 ± 11.9 years, mean BMI was 34.2 ± 6.0 kg/m^2^, mean HbA1c was 7.4 ± 1.1% (57 ± 12 mmol/mol), median duration of diabetes was 61 (32, 120) months, 45% were women and 37% were from a black or minority ethnic group. A total of 18 (7%) subjects had diet-controlled T2D, 128 (52%) were taking a single oral glucose-lowering agent, 64 (26%) were taking two or agents, 17 (7%) were taking three or more oral agents, and 20 (8%) were insulin-treated.

**Table 2.** Demographic, anthropometric, and biochemical characteristics.

T2Ds (*n* = 247)	
Demographics	
Age, years	51.8 ± 11.9
Male, *n* (%)	136 (55)
Female, *n* (%)	112 (45)
Ethinicity	
Caucasian, *n* (%)	155 (63)
Black or other minority ethnicity, *n* (%)	92 (37)
Anthropometrics	
Height, cm	168 ± 10
Weight, kg	96.9 ± 19.1
Body mass index, kg/m^2^	34.2 ± 6.0
Systolic BP, mmHg	138 ± 16
Diastolic BP, mmHg	87 ± 8
Heart rate, beats/min	76 ± 12
Medical history	
Diabetes duration, months	61 (32–120)
Never smoked, *n* (%)	140 (56)
Ex-smoker, *n* (%)	68 (28)
Current smoker, *n* (%)	39 (16)
Hypertension, *n* (%)	121 (49)
Dyslipidaemia, *n* (%)	148 (60)
Medications	
ACE inhibitor, *n* (%)	67 (27)
ARB, *n* (%)	28 (11)
Beta blocker, *n* (%)	16 (6)
Calcium channel blocker, *n* (%)	50 (20)
Statin, *n* (%)	144 (58)
Metformin, *n* (%)	214 (87)
Sulfonylurea, *n* (%)	50 (20)
DPP-IV inhibitor, *n* (%)	16 (6)
SGLT2 inhibitor, *n* (%)	36 (15)
GLP-1 receptor agonist, *n* (%)	17 (7)
Insulin, *n* (%)	20 (8)
Fasting blood tests	
Creatinine, mmol/l	74 ± 16
Estimated GFR, ml/min	84 ± 10
Glucose, mmol/l	7.7 (6.7–9.5)
HbA1c, %	7.4 ± 1.1
HbA1c, mmol/mol	57 ± 12
LDL cholesterol, mmol/l	2.4 ± 0.8

Data are *n* (%), mean ± SD, or median (IQR).

ACE, angiotensin converting enzyme; ARB, angiotensin receptor blocker; BP, blood pressure; DPP-IV, dipeptidyl peptidase-IV; GFR, glomerular filtration rate; GLP-1, glucagon-like peptide-1; IQR, interquartile range; LDL, low-density lipoprotein; SGLT2, sodium glucose co-transporter 2.

### Cardiovascular structure and function

CMR and echocardiography data are displayed in Table 3. Mean LV:mass volume was 0.84 ± 0.14 g/ml, mean LV ejection fraction was 67 ± 7%, mean GLS was −16.2 ± 2.7%, mean MPR was 2.6 ± 1.2, median E/A ratio was 0.84 (0.66, 1.05), and median E/e′ was 7.1 (3.1, 9.4). Overall prevalence of non-ischaemic LGE was low (14%).

**Table 3.** Cardiovascular magnetic resonance and echocardiography data.

T2D (*n* = 247)	
LV end diastolic volume, ml	145 ± 35
LV indexed end diastolic volume, ml/m^2^	68 ± 12
LV end systolic volume, ml	48 ± 18
LV indexed end systolic volume, ml/m^2^	23 ± 7
LV ejection fraction, %	67 ± 7
LV mass, g	119 ± 27
LV mass indexed to height,^7^ g/m^2^	29 ± 5
LV mass:volume, g/ml	0.84 ± 0.14
LV global longitudinal strain, %	−16.2 ± 2.4
Stress myocardial blood flow, ml/g/min	3.11 ± 1.26
Rest myocardial blood flow, ml/g/min	1.17 ± 0.53
Myocardial perfusion reserve	2.60 ± 1.24
Aortic distensibility, mmHg^−1^ × 10^−3^	2.75 (1.74–4.03)
Late gadolinium enhancement present, *n* (%)	35 (14)
E/A ratio	0.84 (0.66–1.05)
Average E/e′	7.1 (3.1–9.4)

LV, left ventricle.

### Correlations of clinical characteristics with cardiovascular structure and function

**Figure 3.**
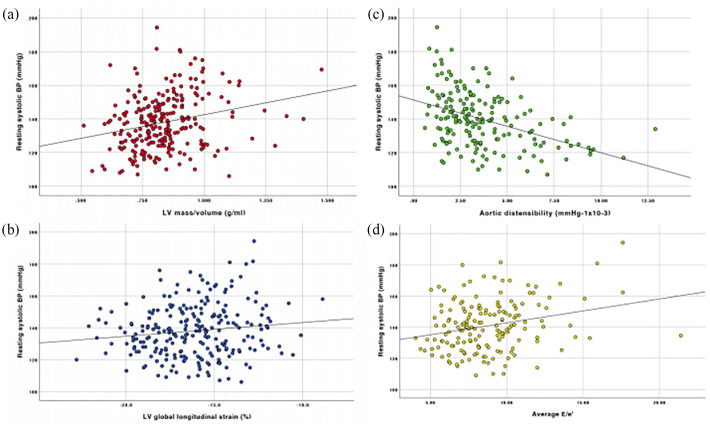
Scatterplots displaying correlations between systolic blood pressure and (a) LV mass:volume, (b) LV global longitudinal strain, (c) aortic distensibility and (d) average E/e′. BP, blood pressure; LV, left ventricle.

Correlations of key CMR and echocardiographic measures of cardiovascular structure and function with patient clinical characteristics are displayed in Table 4 and Figure 3.

**Table 4.** Correlations between clinical characteristics and key measures of cardiovascular structure and function.

	E/A	E/e′	LV mass:vol.	LV GLS	Stress MBF	Rest MBF	MPR	Aortic distensibility
	rho	rho	rho	rho	rho	rho	rho	rho
Age	−0.047	0.531**	0.259**	0.033	−0.657**	−0.588**	−0.207**	−0.302*
T2D duration	0.013	0.389**	0.192*	0.118	−0.332**	−0.322**	−0.107	−0.195*
No. glucose-lowering drugs	0.048	−0.020	0.136*	0.108	−0.062	−0.011	−0.067	0.049
Weight	−0.21	−0.196**	−0.088	0.013	0.258**	0.129	0.099	0.057
BMI	−0.022	−0.141*	−0.079	−0.125*	0.284**	0.286**	−0.089	0.081
Systolic BP	0.016	0.307**	0.252**	0.106	−0.092	−0.096	−0.090	−0.195*
Diastolic BP	−0.016	−0.016	0.192**	0.194**	0.084	0.046	−0.018	−0.052
Creatinine	0.126	0.283**	0.146*	0.129*	−0.217**	−0.375**	0.168**	−0.076
Glucose	−0.055	−0.096	−0.018	0.030	0.017	−0.001	0.057	−0.054
HbA1c	−0.071	−0.161*	−0.001	0.086	0.104	0.092	0.087	−0.003
LDL	<0.001	−0.111	−0.012	−0.117	0.190**	0.113	0.103	0.033

Data shown are correlations coefficients (*r*). *p value < 0.05. **p value < 0.01.

BMI, body mass index; BP, blood pressure; GLS, global longitudinal strain; LDL, low-density lipoprotein; MBF, myocardial blood flow; MPR, myocardial perfusion reserve.

#### Cardiac remodelling and systolic strain

Age (rho = 0.259), duration of T2D (rho = 0.192), number of glucose-lowering agents (rho = 0.136) systolic (rho = 0.252) and diastolic BP (rho = 0.192), and serum creatinine (rho = 0.146) correlated significantly with LV mass:volume. Body mass index (rho = −0.125), diastolic BP (rho = 0.194) and serum creatinine (rho = 0.129) correlated significantly with LV GLS.

#### Diastolic function

No significant correlations were observed between clinical characteristics and E/A ratio. Age (rho = 0.531), duration of T2D (rho = 0.389), body weight (rho = −0.196) and BMI (rho = −0.141), systolic BP (rho = 0.307), serum creatinine (rho = 0.283) and HbA1c (rho = −0.161) all correlated with echocardiographic estimates of LV filling pressure (E/e′).

#### Aortic stiffness

Age (rho = −0.302), duration of T2D (rho = −0.195) and systolic BP (rho = −0.195) were the only significant correlations with aortic distensibility, each of which had an inverse association.

#### MBF and perfusion reserve

Age (rho = −0.657), duration of T2D (rho = −0.332), body weight (rho = 0.258) and BMI (rho = 0.284), serum creatinine (rho = −0.217) and LDL cholesterol (rho = 0.190) were significantly correlated with stress MBF. Similarly, age (rho = −0.588), duration of T2D (rho = −0.322) and BMI (rho = 0.286), correlated significantly with rest MBF. Only age (rho = −0.227) and serum creatinine (rho = 0.168) were significantly associated with MPR.

### Multivariable associations of clinical characteristics with cardiovascular structure and function

Multivariable associations of key clinical parameters with CMR and echocardiographic measures of cardiovascular structure and function are presented in Table 5.

**Table 5.** Multivariable associations between clinical characteristics and cardiovascular structure and function.

	LV mass:volume		GLS		MPR		Aortic distensibility		E/e′	
	β	*p* value	β	*p* value	β	*p* value	β	*p* value	β	*p* value
Age (per 1SD increase)	0.024 (0.001, 0.047)	**0.041**	−0.223 (−0.606, 0.160)	0.254	−0.364, (−0.565, −0.162)	**<0.001**	−0.629 (−1.057, −0.201)	**0.005**	1.273 (0.707, 1.839)	**<0.001**
Male sex	0.008 (−0.032, 0.048)	0.69	0.478 (−0.196, 1.152)	0.164	0.458 (0.107, 0.808)	**0.010**	−0.084 (−0.758, 0.451)	0.807	−1.291 (−2.281, −0.301)	**0.011**
White European ethnicity	−0.016 (−0.054, 0.023)	0.423	−0.04 (−0.68, 0.60)	0.903	0.120 (−0.212, 0.451)	0.479	−0.151 (−0.754, 0.451)	0.622	−0.789 (−1.764, 0.186)	0.112
Never smoked	−0.031 (−0.081, 0.02)	0.233	−1.196 (−2.04, −0.354)	**0.005**	0.197 (−0.233, 0.628)	0.369	0.369 (−0.464, 1.201)	0.383	0.374 (−0.89, 1.637)	0.562
Glucose-lowering agents	0.022 (0.002, 0.042)	**0.030**	0.30 (−0.037, 0.637)	0.081	−0.104 (−0.282, 0.074)	0.253	0.048 (−0.270, 0.366)	0.767	−0.027 (−0.562, 0.509)	0.922
Systolic BP (per 1SD increase)	0.027 (0.001, 0.047)	**0.041**	0.328 (0.019, 0.638)	**0.038**	−0.112 (−0.270, 0.046)	0.166	−0.348 (−0.636, −0.60)	**0.018**	0.735 (0.286, 1.183)	**0.001**
T2D duration (per 1SD increase)	−0.001 (−0.023, 0.020)	0.898	0.262 (−0.10, 0.624)	0.156	0.012 (−0.178, 0.201)	0.902	−0.171 (−0.504, 0.162)	0.314	0.526 (−0.008, 1.06)	0.054
BMI (per 1SD increase)	−0.004 (−0.023, 0.015)	0.689	−0.348 (−0.667, −0.03)	**0.032**	−0.150 (−0.319, 0.019)	0.082	0.089 (−0.257, 0.434)	0.609	0.028 (−0.476, 0.532)	0.913
HbA1c (per 1SD increase)	−0.004 (−0.024, 0.015)	0.678	0.121 (−0.206, 0.448)	0.468	0.087 (−0.087, 0.261)	0.328	−0.017 (−0.316, 0.283)	0.913	−0.296 (−0.809, 0.217)	0.256
Creatinine (per 1SD increase)	0.01 (−0.011, 0.031)	0.333	0.099 (−0.252, 0.45)	0.581	0.140 (−0.047, 0.327)	0.142	0.098 (−0.25, 0.446)	0.577	0.707 (0.172, 1.243)	**0.010**
LDL (per 1SD increase)	0.001 (−0.017, 0.020)	0.892	−0.079 (−0.388, 0.23)	0.617	0.028 (−0.148, 0.205)	0.749	−0.150 (−0.438, 0.138)	0.288	0.071 (−0.401, 0.543)	0.768

BMI, body mass index; BP, blood pressure; GLS, global longitudinal strain; LDL, low-density lipoprotein; LV, left ventricle; MPR, myocardial perfusion reserve. Bold typeface indicates statistical significance *p* < 0.05.

#### Cardiac remodeling

Age (β = 0.024, *p* = 0.041), systolic BP (β = 0.027, *p* = 0.041) and number of glucose-lowering drugs (β = 0.022, *p* = 0.03) were independently associated with LV mass:volume ratio.

#### Diastolic function

Age (β = 1.273, *p* < 0.001), sex (β = −1.291, *p* = 0.011), systolic BP (β = 0.735, *p* = 0.001) and serum creatinine (β = 0.707, *p* = 0.01) were all independently associated with average E/e′.

#### Global longitudinal strain

Smoking history (never smoked β = −1.196, *p* = 0.005), systolic BP (β = 0.328, *p* = 0.038) and BMI (β = −0.348, *p* = 0.032) were independently associated with GLS.

#### Myocardial perfusion reserve

Age (β = −0.364, *p* < 0.001) and sex (β = 0.458, *p* = 0.01) were the only independent associations with MPR.

#### Aortic distensibility

The variables associated independently with aortic distensibility were age (β = −0.629, *p* = 0.005) and systolic BP (β = −0.348, *p* = 0.018).

## Discussion

This is the first study to explore clinical determinants of early diabetic cardiomyopathy in a large, multi-ethnic cohort of asymptomatic adults with T2D who were phenotyped comprehensively with both multiparametric CMR and echocardiography. The major clinical determinants of markers of SBHF in our cohort were increasing age, systolic BP, BMI, serum creatinine and smoking history.

Contrary to some reports, neither HbA1c nor duration of diabetes were associated with any measure of cardiovascular function in our multivariable analyses, although there was a modest correlation between HbA1c and GLS. The lack of association between glycaemic control and measures of diastolic and systolic dysfunction is perhaps not surprising given the lack of evidence to suggest that improved glycaemic control lowers incident heart failure in people with T2D.^18^ Our findings contradict others, where both diastolic and systolic function have been found to worsen across the glycaemic spectrum.^19,20^ However, these were much smaller studies than ours (comprising less than 100 subjects with T2D) and only utilised echocardiography, with significant risk of overfitting regression analyses. We did find a modest association between the number glucose-lowering therapies and LV mass:volume, which could indicate a weak link between severity of dysglycaemia and concentric LV remodelling. However, the number of diabetes drugs was not associated with any other measure of cardiovascular function. Overall these findings suggest that glycaemic control is not itself a central mechanism driving heart failure in T2D.

Increasing systolic BP was associated independently with more concentric LV remodelling, worse GLS and E/e′, and lower aortic distensibility. The associations between T2D, BP, arterial stiffening and LV hypertrophy are well described,^21,22^ and these relationships are confirmed in our own cohort. Hypertension has been shown to cause reductions in GLS (especially in patients with long-standing disease),^23^ is associated with a higher prevalence of diastolic dysfunction in asymptomatic individuals and is a recognised risk factor for HFpEF.^24,25^ Co-existence of T2D and hypertension confers a greater risk of cardiovascular disease compared with either diabetes or hypertension in isolation.^26^ Intensive BP reduction, however, does not appear to lower the risk of incident heart failure (although overall mortality, cardiovascular death, myocardial infarction and stroke rates do improve with tighter BP control).^27^ As with T2D duration, perhaps earlier, aggressive BP reduction in younger adults with T2D at highest risk may be required to halt progression of subclinical cardiac dysfunction and prevent heart failure development. Similarly, a history of never having smoked was strongly associated better GLS, potentially supporting a role for smoking cessation to prevent or reduce systolic dysfunction in people with T2D, amongst other cardiovascular benefits.

The other marker of metabolic disease examined in this study – BMI – was associated with “higher” GLS (described by an inverse regression coefficient). This suggests that increasing BMI leads to hyperdynamic LV function. It is recognised that obesity is associated with increased sympathetic activity, which may result in hyperdynamic LV function.^28^ Weight loss with a 12-week low-energy diet has been suggested recently to reduce supra-normal LV ejection fraction in people with T2D,^6^ which may represent normalisation of hyperdynamic LV function, although further studies are needed to confirm the effects of weight loss on GLS.

Lastly, we found in our T2D patients that only increasing age and female sex were associated with lower MPR. Although microvascular dysfunction is thought to be a key determinant of diabetic cardiomyopathy, none of our modifiable clinical risk factors were significantly associated with MPR in multivariable analysis. Two previous studies have reported correlations between fasting glucose and HbA1c, but both were limited by small sample sizes (*n* = 25 and *n* = 23) preventing multivariable analyses.^29,30^ The metabolic determinants of microvascular dysfunction and the relationship between MPR and cardiac systolic and diastolic dysfunction in T2D, therefore, warrant further investigation. Indeed, we have recently shown that MPR is associated with aerobic exercise capacity in asymptomatic adults with T2D, independent of age, sex, ethnicity, smoking status, glycaemia and diastolic function.^5^ This suggests a central role for microvascular dysfunction in SBHF in people with T2D.

### Limitations

Although this is amongst the largest studies examining asymptomatic adults with T2D who have been extensively phenotyped with CMR and echocardiography, the overall sample size remains modest. The pooled cohort of subjects from four separate studies of cardiac structure and function in our unit is a limitation, owing to minor differences in inclusion criteria. However, we adhered to pre-specified inclusion and exclusion criteria for these pooled analyses to limit heterogeneity of included subjects. Furthermore, all CMR scanning and echocardiography was performed standardised protocols across all four studies, to minimise the risk of heterogeneity in sampling. Although all CMR scanning utilised a standardised imaging protocol, the impact of different MRI field strengths on perfusion is not known. This may have impacted absolute stress and rest MBF values but should not significantly affect MPR. Although we excluded significant epicardial coronary artery disease by LGE imaging and stress perfusion CMR,^31^ invasive coronary angiography remains the gold-standard technique for assessment of coronary disease and individuals with significant diffuse three-vessel disease may not have been detected by CMR imaging alone.

## Conclusion

In a large, asymptomatic, multi-ethnic cohort of adults with T2D, the major clinical determinants of cardiovascular dysfunction were increasing age, duration of T2D, systolic BP, BMI and smoking history. HbA1c was not associated with early heart failure. Whether early interventions to treat modifiable risk factors (such as weight loss, BP reduction, reversal of T2D and smoking cessation) improve subclinical cardiovascular dysfunction is not known.
